# Association of Craniofacial Patterns with the Curve of Spee and the Time Required for Orthodontic Levelling

**DOI:** 10.3390/dj10090175

**Published:** 2022-09-16

**Authors:** Annina Krüsi, Konstantinos Dritsas, Eleni Kalimeri, Dimitrios Kloukos, Nikolaos Gkantidis

**Affiliations:** 1 Department of Orthodontics and Dentofacial Orthopedics, School of Dental Medicine, University of Bern, CH-3010 Bern, Switzerland; 2 Department of Orthodontics and Dentofacial Orthopedics, 251 Hellenic Air Force and VA General Hospital, GR-115 25 Athens, Greece

**Keywords:** prospective cohort study, treatment time, fixed orthodontic treatment, levelling, alignment, curve of Spee

## Abstract

The curve of Spee (CoS) is an important parameter for an individualized treatment plan. The available information regarding a potential association of the depth of the curve of Spee with various skeletal craniofacial characteristics is conflicting and it is also unknown whether certain craniofacial parameters affect the duration of the levelling phase of orthodontic treatment. A prospective sample of 32 patients with mild to moderate crowding that underwent orthodontic treatment with full fixed appliances was used to study these topics. The craniofacial characteristics were captured on pre-treatment lateral cephalometric radiographs and measurements of the CoS were performed on the initial 3D digital dental models using the Viewbox 4 software. Non-parametric statistics and Spearman’s correlations were applied. Weak negative correlations were detected between the CoS depth and the SNA and SNB angles. There was no other association between the CoS and craniofacial parameters, including various anteroposterior measurements. Furthermore, there was no significant association of any craniofacial parameter with the duration of the levelling. Contrary to certain clinical beliefs, it can be argued that the craniofacial characteristics are not associated with the CoS and the time required for its levelling in subjects with moderate pre-treatment CoS depth.

## 1. Introduction

The curve of Spee (CoS) is one of the main characteristics of the dentition that are considered during the establishment of a treatment plan and is defined as the anteroposterior curvature of the mandibular and maxillary dentition [1,2]. After the primary dentition, the CoS has a natural tendency to deepen over time, but it remains relatively stable in adulthood [3]. The development of the CoS during growth might be affected by multiple factors, such as the timing of the eruption of the permanent teeth, the masticatory forces and the overall development of the craniofacial system [3,4].

A flat or a slight CoS is a key objective of an orthodontic treatment as suggested by Andrew’s 6 keys for a normal occlusion [5] and is achieved primarily through the extrusion of the lower premolars, and to a lesser extent the intrusion of the lower incisors [6]. The orthodontic CoS correction is reported to be relatively stable in the long-term [7]. Still, studies have shown that the tooth movements occurring during the levelling of the CoS vary among different vertical skeletal patterns [8,9], which also show different long-term stability after treatment [10]. Brachyfacial patients with increased deep bite were more prone to relapse, in contrast to dolichofacial patients [10,11].

So far, there is conflicting information available in the literature regarding a potential association of the depth of the CoS with various craniofacial characteristics. Farella et al. found a negative association of the CoS with the SNB angle, which was not in accordance with the findings of Halimi et al., who did not detect any association [12,13]. This could be attributed to the heterogeneity of the exact methods that are present in the literature. In some cases, error prone methods are utilized, such as the use of a camera [12], which might be affected by lens distortion. The definition of the CoS also varies across studies, as it is either defined only by the tooth cusps [9] or extended to the contact points [10,13] and the distances were either averaged or only the maximum ones were considered. Furthermore, we did not identify any study testing the association of craniofacial skeletal pattern to the duration of the mandibular arch levelling. It might be expected that the levelling phase requires more time in low angle patients due to the associated differences in functioning (stronger masticatory system, higher occlusal forces, etc.) [14,15,16,17] and the required tooth movements [8,9,18].

Thus, the aim of this study was to investigate the association of (a) the depth of CoS, expressed both as the average and the maximum distance of the tooth cusps to the occlusal plane, and (b) the time required for CoS levelling, with various sagittal and vertical craniofacial patterns of patients treated with fixed orthodontic appliances in both jaws. We hypothesized that brachyfacial patients have deeper CoS and require more time for CoS levelling.

## 2. Materials and Methods

### 2.1. Sample

This was a prospective observational study, which included all patients originating from a randomized controlled trial, investigating the effect of the timing of the second molar bonding on the duration of the CoS levelling phase [19]. In the previous publication, the sample was divided into two groups according to the timing of the second molar bonding; at the start of the treatment or before the insertion of the second archwire (0.016″ × 0.022″ NiTi). The study did not show any significant difference, thus, the sample can be considered as one homogenous group for further testing.

The sample for this study consisted of patients who underwent orthodontic treatment at the Department of Orthodontics and Dentofacial Orthopedics of the 251 Greek Air Force Hospital, between January 2015 and July 2017. 

All patients included in this study fulfilled the following criteria:

- Adolescent patients of any sex between 12–18 years of age
- Fully erupted permanent teeth, except for the third molars
- Absence of periodontal or any systemic disease
- Non-extraction treatment with full fixed appliances
- Absence of mechanics that require bands on molars
- Maximum overall crowding of 5 mm

### 2.2. Interventions

As reported previously [19], all patients received treatment with full fixed self-ligating appliances using the following archwire sequence: 0.014″ Sentalloy 80 gr (NiTi), 0.016″ × 0.022″ Neo Sentalloy 80 gr (NiTi), and 0.017″ × 0.025″ stainless steel (SS) (Dentsply GAC. Islandia, New York, NY, USA). The fixed appliances were directly bonded at the same appointment in both jaws using the Transbond-XT resin (3M Unitek, Monrovia, CA, USA). To eliminate any occlusal interference, the bite was raised posteriorly with resin-modified glass ionomer cement material (Ultra Band-Lok^®^, Reliance, IL, USA) on the palatal cusps of the upper second molars, allowing 1 mm of space between antagonists. For consistency reasons, patients without any interferences after bonding also received bite openings of 1 mm. 

The time point of the completion of the levelling phase was defined as the day on which the insertion of the 0.017″ × 0.025″ SS wire could be accomplished. The patients were monitored at one-month intervals under the supervision of a single experienced specialist (Dimitrios Kloukos).

### 2.3. Measured Variables

As reported previously [19], pre-treatment plaster models were acquired through alginate impressions (Tetrachrom Alginat, KANIEDENTA GmbH & Co. KG, Zum Haberland 36, Herford, Germany), and were cast with plaster (Alabaster Klasse 3, Wiegelmann Dental GmbH, Landsberger Strasse 6, Bonn, Germany) within the same day. The models were digitised afterwards with an intraoral scanner (TRIOS 3, 3Shape, Copenhagen, Denmark, Software Version 1.4.7.5), to generate the Standard Tessellation Language (STL) models for the measurement of the CoS.

**Figure 1:**
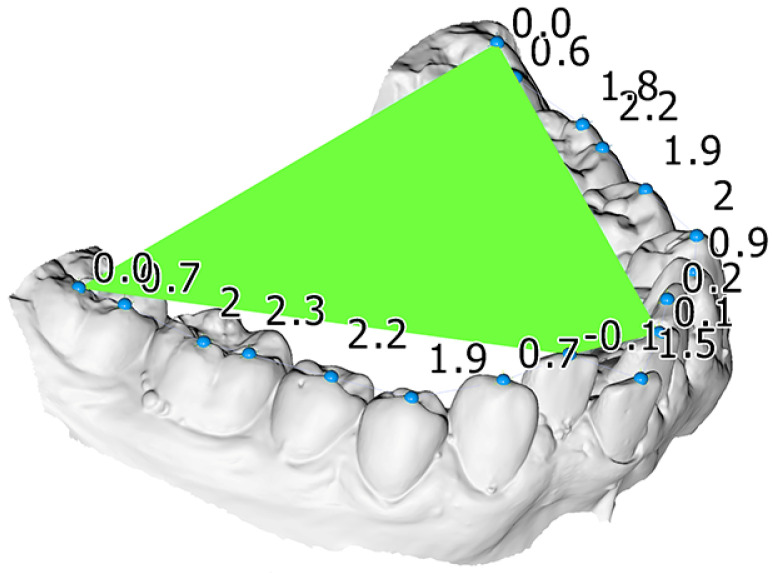
Mandibular dental model of a patient included in this study. A best fit occlusal plane (green), constructed through landmarks placed on each tooth (blue spheres), was used to measure the curve of Spee. The numbers indicate the vertical distance of each tooth cusp landmark from this plane, used to calculate the average and the maximum curve of Spee measurement.

One operator (KD) performed the measurements on the digital models using the Viewbox software (Viewbox 4 software, dHAL Software, Kifisia, Greece). Crowding was measured on the digital models by measuring the difference between tooth width and the available space for each misaligned tooth. To measure the CoS, landmarks were placed on each tooth cusp and a best-fit plane to the landmarks corresponding to the most occlusally located molar cusps and incisal edges of each side served as the occlusal plane. Afterwards the distances of the landmarks to the occlusal plane were calculated (Figure 1). The average CoS was defined as the mean of all distances to the occlusal plane and the maximum of these distances comprised the maximum CoS measurement. The process is described in more detail in the study by Dritsas et al. [19].

**Figure 2:**
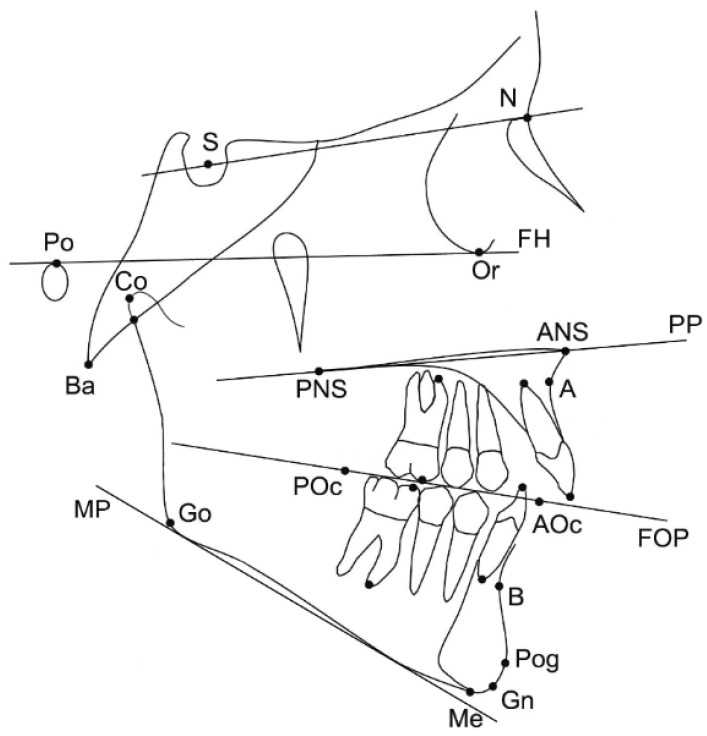
Landmarks and reference planes that were used for the study. Skeletal landmarks: sella (S), nasion (N), porion (Po), orbitale (Or), anterior nasal spine (ANS); posterior nasal spine (PNS); A-point (A), B-point (B), pogonion (Pog), gnathion (Gn), menton (Me), gonion (Go), condylion (Co), basion (Ba). Dental landmarks: maxillary and mandibular first molar mesial cusp, maxillary and mandibular first molar mesial apex, maxillary and mandibular incisor tip, maxillary and mandibular incisor apex, posterior occlusal point (POc), anterior occlusal point (AOc) (occlusal points were placed arbitrarily along the functional occlusal plane based on the occlusal contacts of the premolars and molars). Reference planes: Frankfurt horizontal plane (FH), palatal plane (PP), functional occlusal plane (FOP), mandibular plane (MP) (line tangent to the lower part of the mandible).

The pre-treatment lateral cephalograms were obtained within one month before the treatment started. Twenty-five landmarks on dental and skeletal structures were digitised on screen by one experienced operator (NG), using the Viewbox 4 software. All X-rays were of adequate diagnostic quality and were adjusted for magnification by using the included reference ruler. Fourteen cephalometric measurements (7 angular, 5 linear and 2 ratios) were selected for the cephalometric analysis (Figure 2).

### 2.4. Statistical Analysis

The study variables were tested for normality through the Shapiro-Wilk test and few variables showed deviation from normality. Thus, non-parametric statistics were applied in the study.

Bivariate Spearman’s correlations were performed between selected cephalometric variables that depicted craniofacial form, as well as between the amount of average CoS, maximum CoS and the days required for levelling. The level of significance was set at 0.05.

### 2.5. Method Error

The cephalometric measurements were repeated by the same investigator in twelve randomly selected cases, following a two-week period. Dahlberg’s error formula was used to calculate the estimated error, and the differences were tested for significance pairwise with the Wilcoxon signed-rank test.

## 3. Results

### 3.1. Participants

A total of 32 patients (19 males and 13 females) were included in this study with a median age of 14.9 years (range: 12.1–18.3 years) at the time of the bonding. The sample’s pre-treatment characteristics as well as the days required to level the CoS are presented in Table 1.

### 3.2. Main Outcomes

**Figure 3:**
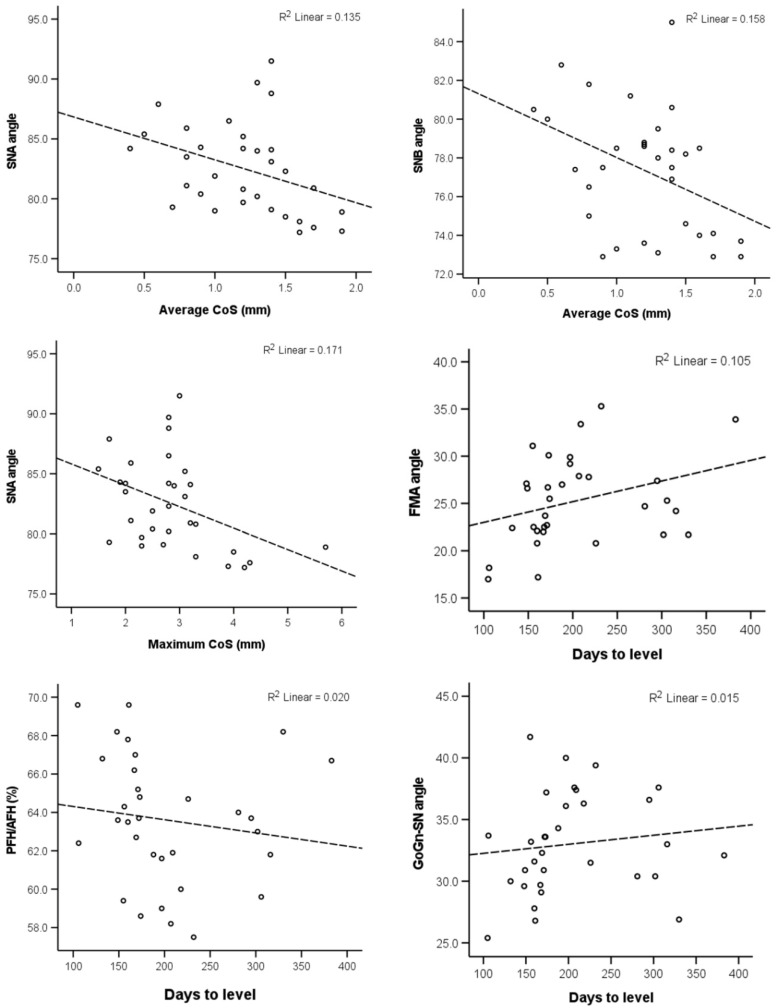
Scatter plots depicting the relevant data regarding the correlations reported in Table 2 and had *p* < 0.10. The dashed lines show the least squares linear lines that best fit the data.

The correlation of the CoS severity and the days required to level it with the selected craniofacial measurements are presented in Table 2. The null hypothesis was rejected, since there was no evidence that brachyfacial patients have deeper CoS and require more time for CoS levelling. The maximum CoS value was found to have a weak negative association with the SNA value. The average CoS also exhibited a weak negative association with the SNA, as well as the SNB angle. Finally, the duration of the levelling was weakly correlated positively to the FMA angle (*p* < 0.05). Scatter plots of the statistically significant correlations, as well as any correlations with *p* < 0.1, are presented in Figure 3.

### 3.3. Method Error

The Dahlberg’s error rates are presented in Table A1 and are considered acceptable. No statistically significant difference was detected for any measurement. The ANB, GoGn-SN and PFH/AFH variables approached the level of significance, but showed median differences of 0.45 mm, 0.95° and 1.6%, respectively, which were relatively small, without any clinical significance.

## 4. Discussion

The evaluation of a patient’s pre-treatment characteristics is an important consideration for an individualized treatment plan, since it may allow us to anticipate a patient’s response to certain treatment modalities. The present study investigated the association of various craniofacial patterns with the CoS and the days required to level it and detected only a few weak correlations. Thus, based on the current findings, we were not able to identify concrete predictive factors regarding the levelling duration or verify certain clinical beliefs, such as the association of the CoS to vertical craniofacial parameters. The predictive capacity of these factors was not confirmed for patients with similar characteristics to those included here and should not affect treatment decisions.

A weak correlation was detected between the CoS and two sagittal craniofacial measurements, namely the SNA and SNB angle. According to our analysis, an increase in the average depth of the CoS is weakly associated with a more posteriorly positioned maxilla or mandible. A similar relationship was detected between the maximum CoS depth and the SNA angle. However, no association was found between the CoS and the maxillary or mandibular length or the ANB value and the aforementioned correlations explained only about 20% of the observed variance. Farella et al. also detected a negative relationship between the SNB angle and the CoS curvature, but not the SNA angle [12]. Furthermore, our findings are in contrast with the study of Halimi et al. [13], who did not find any such correlation. Therefore, a meaningful correlation between the CoS and the sagittal craniofacial measurements cannot be supported at present.

It is a common clinical belief that the CoS is related to the vertical dimension, with a brachyfacial craniofacial pattern being associated with a deeper CoS and an increased overbite. Our data, which are representative of the average vertical variation in the population, cannot corroborate this argument, and this is also in agreement with other similar studies [8,12,13]. Perhaps such an association could have been established if extreme vertical patterns were compared to each other, but this remains to be tested.

A possible association of the time required to level the CoS with certain craniofacial patterns would have major clinical significance, because it would contribute to a more accurate estimation of the treatment duration, which is an important factor affecting patient’s satisfaction [20] and compliance [21] and could enhance the doctor-patient communication. Moreover, the establishment of a valid predictive factor for the treatment duration, would allow for targeted treatment plans in regard to possible iatrogenic effects related to orthodontic treatment duration, such as white spots or root resorption [22,23], and thus, could enhance patient compliance and satisfaction [24,25]. Our study identified a weak positive relationship between the days to level the CoS and the FMA angle at borderline significance. However, the manual removal of one detected outlier after exploratory testing, did not provide a statistically significant result.

Regarding the sagittal measurements in relation to the levelling duration, no correlation was detected, despite its moderate association with the amount of overjet, as reported by our previous study [19]. This indicates that the levelling duration is dependent mostly on the configuration of the dental structures and not the general craniofacial characteristics. Although the ANB angle has been associated with an increased treatment duration in the past, these studies assessed the total treatment duration [26,27].

According to our previous study [19], contrary to common clinical belief, the amount of mild to moderate crowding present in our sample was not found to affect CoS levelling duration, and thus, the crowding factor was not further investigated in the present study. This may not hold true for patients with a more pronounced CoS or more severe crowding, and should be further investigated. Overall, there is a lack of evidence in the literature regarding the duration of the levelling phase. A possible reason is the plethora of treatment strategies and clinicians’ preferences for this purpose, which reduces the generalizability of study findings. Furthermore, the optimal endpoint of the levelling phase is hard to pinpoint. We selected the 0.017″ × 0.025″ stainless steel wire, as it is a commonly used wire, and the CoS must be relatively flat for it to be inserted.

### Limitations

The main limitation of this study could be the moderate sample size. With a larger sample, it may have been possible to verify or refute some of the few correlations that approached but did not reach clinical significance. However, the correlation coefficients were low in all cases. Another limitation is that the sample had mild to moderate crowding and average CoS depth, which are factors that could potentially influence the levelling duration if they were more severe. Furthermore, the present study included non-extraction cases, and thus, future studies should also test if the presence of tooth extractions or missing teeth during levelling modify these outcomes. Finally, in the present study, the end of the levelling phase was when the clinical decision to insert the 0.017″ × 0.025″ SS wire was taken. In certain cases, a small amount of residual CoS might have been present at that point, as dictated by the bracket play or the small range of vertical activation that the 0.017″ × 0.025″ SS wire allows. Unfortunately, we did not obtain dental models at this time point, and thus, it is not possible to measure any remaining CoS at the endpoint of levelling. However, if present, it would be of a small amount and randomly distributed in the patient sample, and thus, it is not expected to affect the outcomes.

## 5. Conclusions

Based on the current prospective observational study, it can be argued that the craniofacial characteristics are not associated with the amount of CoS and the time required for its orthodontic levelling in subjects with mild to moderate pre-treatment CoS depth and crowding. Only a few weak correlations were detected. Further research could test samples of bigger size, with deeper CoS and more severe crowding.

## Figures and Tables

**Table 1:** Descriptive statistics of all variables analysed in the study.

Variable	Median	IQR *	Range
Max. CoS (mm)	2.8	1.1	1.5–5.7
Average CoS (mm)	1.3	0.6	0.4–1.9
Overjet (mm)	4.0	1.0	1.9–6.9
Overbite (mm)	3.7	1.5	1.5–9.9
Days required to level	173.5	70.5	105–383
SNA (°)	82.1	5.8	77.2–91.5
SNB (°)	77.8	5.3	72.9–85.0
ANB (°)	5.5	2.5	–0.4–10.2
Wits appraisal (mm)	1.9	3.8	–3.5–7.4
Facial angle (FH-NPog) (°)	88.0	2.6	80.8–94.2
FH-MP (FMA) (°)	25.0	5.9	17.0–35.3
MP-PP (°)	20.8	7.4	11.7–29.8
GoGn-SN (°)	32.7	6.4	25.4–41.7
LFH/TFH (%)	54.5	3.6	46.3–60.1
PFH/AFH (%)	63.7	4.9	57.5–69.6

* IQR: Interquartile range, CoS: Curve of Spee.

**Table 2:** Evaluation of the relationship between the CoS and the days to level the CoS with the selected craniofacial measurements.

Variable	Max CoS r (P)	Average CoS r (P)	Days to Level r (P)
SNA (°)	−0.441 (0.011) *	−0.470 (0.007) *	0.128 (0.485)
SNB (°)	−0.282 (0.117)	−0.374 (0.035) *	0.060 (0.744)
ANB (°)	−0.145 (0.427)	−0.074 (0.688)	0.193 (0.290)
Wits appraisal (mm)	0.049 (0.791)	0.112 (0.543)	−0.126 (0.491)
Facial angle (FH-NPog) (°)	−0.133 (0.469)	−0.133 (0.467)	−0.165 (0.367)
FH-MP (FMA) (°)	0.146 (0.424)	0.083 (0.651)	0.350 (0.050) *
MP-PP (°)	0.139 (0.446)	0.137 (0.453)	0.294 (0.102)
GoGn-SN (°)	0.144 (0.431)	0.187 (0.304)	0.306 (0.089)
LFH/TFH (%)	0.079 (0.668)	0.175 (0.339)	0.233 (0.199)
PFH/AFH (%)	−0.210 (0.248)	−0.199 (0.275)	−0.321 (0.073)
Maxillary length (Co-ANS) (mm)	0.061 (0.742)	0.178 (0.330)	−0.041 (0.822)
Mandibular length (Co-Gn) (mm)	0.149 (0.416)	0.250 (0.167)	0.077 (0.674)

* IQR: Interquartile range, CoS: Curve of Spee, r: Spearman’s rank correlation coefficient.

## Data Availability

The data presented in this article are available on reasonable request from the corresponding author.

## Appendix A

**Table A1:** Dahlberg error rates for selected measurements (intraoperator error).

Variable	Dahlberg’s Error	*p*-Value * (Wilcoxon Signed Rank Test)
SNA (°)	0.7	0.432
SNB (°)	0.4	0.288
ANB (°)	0.6	0.050
Wits appraisal (mm)	0.8	0.306
Facial angle (FH-NPog) (°)	0.8	0.271
FH-MP (FMA) (°)	0.9	0.593
MP-PP (°)	0.7	0.209
GoGn-SN (°)	1.0	0.031
LFH/TFH (%)	0.5	0.844
PFH/AFH (%)	1.5	0.023

* *p* < 0.005, Bonferroni correction applied.
